# Dipeptidyl peptidase-4 levels are increased and partially related to body fat distribution in patients with familial partial lipodystrophy type 2

**DOI:** 10.1186/s13098-017-0226-0

**Published:** 2017-04-24

**Authors:** Cynthia Melissa Valerio, Juliana Severo de Almeida, Rodrigo Oliveira Moreira, Luiza Barreto. S. Aguiar, Priscila O. Siciliano, Denise P. Carvalho, Amelio F. Godoy-Matos

**Affiliations:** 1 Metabolism Unit, Instituto Estadual de Diabetes e Endocrinologia (IEDE), Rua Moncorvo Filho 90, Rio de Janeiro, RJ CEP 20211-340 Brazil; 2 Endocrine Physiology Laboratory, Biophysics Institute of Carlos Chagas Filho, Universidade Federal do Rio de Janeiro, Rua Carlos Chagas Filho, 373, Prédio do CCS, Ilha do Fundão, Rio de Janeiro, RJ CEP 21944-97 Brazil

**Keywords:** Dipeptidyl peptidase-4, Lipodystrophy, Adipokines

## Abstract

**Background:**

Dipeptidyl peptidase-4 (DDP4) is an enzyme responsible for glucagon-like peptide-1 inactivation and plays an important role in glucose metabolism.

**Objective:**

The aim of this study was to evaluate DPP4 levels in patients with familial partial lipodystrophy type 2 (FPLD2) and correlate it with body fat distribution.

**Methods:**

Fourteen patients with FPLD2 were selected to participate in this study and matched to a healthy control group (n = 8). All participants had anthropometrical data registered. Body adiposity index (BAI) was used to evaluate fat distribution in this population. Body fat content and distribution were analyzed by dual X-ray absorptiometry (DXA). Biochemical exams, including DPP4 levels, were performed in all individuals.

**Results:**

Despite the same body mass index, lipodystrophic patients had a significant lower hip (median 92.0 vs 94.5; p = 0.028), HDL cholesterol (42.6 ± 10.4 vs 66.1 ± 16.0; p < 0.01) and BAI (24.1 ± 2.8 vs 29.0 ± 3.7; p = 0.02), suggesting that BAI was able to catch differences in fat distribution between groups. On the other hand, patients with FPLD2 presented significant higher levels of insulin (median 11.2 vs 5.3; p = 0.015), triglycerides (184.9 ± 75.4 vs 89.1 ± 51.0; p < 0.01) and DPP4 (4.89 ± 0.92 vs 3.93 ± 1.08; p = 0.04). A trend toward an inverse statistical significance was observed between DPP4 levels and BAI (r = −0.38; p = 0.072). In the lipodistrophic group, a significant correlation was found between DPP4 levels and percentage of total body fat (r = 0.86; p = 0.0025) and android fat (r = 0.78; p = 0.014).

**Conclusions:**

Patients with FPLD2 exhibit an increase in DDP4 levels in comparison to a healthy control group. The increase in the levels of this enzyme does not seem to be related to the diagnosis of diabetes and might be associated with an increase in central fat (estimated using BAI and measured using DXA). These results might be used to reinforce the concept that DDP4 is an adipokine related to central fat distribution.

## Background

Dipeptidyl peptidase-4 (DPP4) is a ubiquitous enzyme knowing to cleave several peptides, including glucagon-like peptide-1 (GLP-1) and gastric inhibitory polypeptide (GIP) [1]. GLP1 and GIP are released from the intestinal mucosa and respond for ~60% of postprandial insulin secretion, also known as incretin effect [2]. DPP4 inhibitors increase active GLP-1 and GIP, modulating pancreatic α and β-cell function, and therefore are part of the target therapeutics for type 2 diabetes [2, 3].

Recently, Lamers et al. [4] demonstrated that DPP4 is expressed and released by mature adipocytes, suggesting that it may be a new adipokine. In an in vitro experiment, DPP4 inhibited insulin-induced Akt phosphorylation, thus linking DPP4 levels to insulin resistance (IR). Furthermore, these authors demonstrated that plasma DPP4 levels were increased in obese patients and were directly related to BMI, leptin and fasting insulin levels, and negatively to adiponectin [4]. Moreover, in adipocytes obtained by biopsy from obese patients, DPP4 expression was five times more evident in visceral in comparison to subcutaneous fat [4]. This relationship between DDP-4 and adipose tissue was re-examined by Sell et al. [5] and similar results were yielded.

Adipokine levels may be altered in states of excessive or decreased body adiposity. We have been studying lipodistrophic patients trying to characterize how adipokines behave in such model of adipose tissue scarcity. In these patients, leptin and retinol binding protein 4 (RBP4) levels seem to be decreased, but have not related with indexes of IR [6].

Familial partial lipodystrophy type 2 (FPLD2) is an inherited disease characterized by lack of peripheral adipose tissue and fat deposition in ectopic sites, such as liver and muscle, which is associated with IR and diabetes [7]. As an adipokine, DPP4 levels may be altered in such models. To the best of our knowledge, there is no published data on DPP4 and FPLD2. Therefore, the aim of this study is to evaluate DPP4 levels in patients with FPLD2.

## Methods

Fourteen patients with FPLD2 were selected to participate in this study. FPLD2 diagnosis was confirmed by molecular analysis of LMNA gene (ABI Prism 3100 Genetic Analyzer, Applied Biosystems, Foster City, CA) provided by the Molecular Endocrinology Laboratory of Paulista Medical School. All patients had a missense mutation in LMNA gene: ten patients harbored the heterozygous variation p.R482W; in three patients, the identified mutation was p.R482Q (c.1445G > A) and one patient exhibited a novel heterozygous variant in exon 8. A control group (two healthy men and six healthy women) was carefully and sequentially selected in order to match the lipodystrophic group by body mass index (BMI) and age. A written informed consent was obtained from all participants after procedures involved in the study were fully explained. The ethic committee of the institution approved the protocol.

Exclusion criteria were as follows: current use or in the last six months of DPP4-inhibitors or GLP-1 agonists, end stage kidney disease, liver failure and heart failure above stage II Functional classification of the New York Heart Association (NYHA).

### Anthropometrical examination

All participants had the following anthropometrical data registered: body weight (Kg), height (m), waist circumference (WC), and waist-to-hip ratio (WHR). BMI was calculated as weight in kilograms divided by the square of height in meters (kg/m^2^). Waist circumference was determined at the midpoint between the lowest rib and the iliac crest. WHR was defined as the ratio of waist girth to the largest circumference of the hips, measured at the trochanter major. Body adiposity index (BAI) was calculated according to the formula proposed by Bergman et al. [8]: ${\text{BAI}} = \,\left( {{\text{Hip}}/{\text{Height}}^{1.5} } \right) - 18.$

### Body fat analysis

Dual X-ray absorptiometry (DXA) scan (LUNAR PRODIGY ADVANCE software version 9.5, LNR 41569 model; GE Medical Systems, Waukesha, WI) was used to estimate human body fat content. Fat quantity and distribution were quantified in percentage (%): total and troncular fat, upper and lower limbs fat, android and gynoid fat mass. Whole body DXA scans were obtained using manufacturer’s recommendations for subject positioning, scan protocols, and scan analysis.

### Laboratory evaluation

Routine laboratory tests were performed by CientificaLab Laboratory: fasting plasma glucose (FPG—enzymatic colorimetric method), Hemoglobin A1c [HbA1c—high performance liquid chromatography (HPLC)], cholesterol and fractions (Enzymatic colorimetric) and insulin (electrochemiluminescence). DPP4 was measured utilizing a commercial ELISA kit of dipeptidyl Peptidase IV (E90884Hu) USCN Life Science Inc. Houstoun, USA. This measurement was conducted with the support of the University of Rio de Janeiro (Endocrine Physiology LaboratoryCarlos—Chagas Filho Institute).

Insulin resistance was evaluated by HOMA-IR (homeostasis model assessment) calculated by the formula [Insulin (mU/L) × Glucose (mmol/L)/22.5] and ß-cell function (HOMA-β) by the formula [insulin (mU/L) × 20/Glucose (mmol/L) − 3.5)]. Insulin sensitivity was evaluated by QUICKI (quantitative insulin sensitivity check inbox) calculated by the formula [1/(log Insulin Glucose + log)].

### Statistical analysis

Statistical analysis was performed with GraphPad InStat 3.00 for Windows 95 (GraphPad Software, San Diego, California, USA). Kolmogorov–Smirnov test was used to identify non-parametric variables. The unpaired t test was used for parametric variables and Mann–Whitney for non-parametric variables. The strength of the linear relationship between two continuous variables was evaluated by means of the Pearson’s correlation coefficient or Spearman’s correlation coefficient. The level of statistical significance was 5%.

## Results

Fourteen women with FPLD2 were evaluated in our study. Patients belonged to seven different families and have been previously characterized [9]. In brief, six patients had the diagnosis of lipoatrophic diabetes with average duration of 10.8 years since diagnosis. Three of them were in use of metformin, one was using sulphonylurea and one was on insulin and pioglitazone therapy. None of the patients in the lipodistrohic group were using any medications that could affect DPP4 levels, as incretinomimetic agents. Table 1 presents the comparison between control group and lipodystrophic subjects. None of the subjects included in control group had diabetes. Despite the same age and BMI, lipodystrophic patients had significant differences in insulin sensitivity (insulin, HOMA-IR and QUICKI), anthropometric (i.e. hip) and metabolic parameters (i.e. HDL cholesterol and triglycerides). For the same BMI, BAI was able to catch differences in total body fat content between groups. DPP4 was increased in patients with FPLD2 in comparison to the control group.

**Table 1:** Clinical and biochemical variables in a sample of patients with familial partial lipodystrophy type 2 (FLPD2) in comparison to control group

	FLPD (n = 14)	Control (n = 8)	P value
Age (years)	40.0 ± 12.6	31.3 ± 17.7	0.19
Gender (female)	12	6	0.60
Weight (kg)	66.2 ± 10.0	60.1 ± 8.8	0.16
BMI (kg/m^2^)	24.1 18.5–26.9	23.4 19.2–27.9	0.40
Waist (cm)	81.8 ± 8.0	80.7 ± 10.7	0.79
Hip (cm)	92.0 79–96	94.5 89–100	0.028
WHR	0.91 ± 0.06	0.84 ± 0.10	0.08
BAI	24.1 ± 2.8	29.0 ± 3.7	0.002
Glucose (mg/dL)	90.5 70.0–184.0	89.0 81–101	0.56
Insulin (mU/L)	11.2 4.8–42.9	5.3 3.1–13.3	0.015
HOMA-ß	40.4 ± 19.2	21.4 ± 11.9	0.04
HOMA-IR	2.2 1.1–18.7	1.1 0.6–3.3	0.025
QUICKI	0.32 ± 0.03	0.37 ± 0.03	0.02
HDL (mg/dL)	42.6 ± 10.4	66.1 ± 16.0	<0.01
Triglycerides (mg/dL)	184.9 ± 75.4	89.1 ± 51.0	<0.01
HbA_1c_ (%)	5.6 5.2–9.1	5.4 4.8–6.1	0.14
DPP-4 (ng/mL)	4.89 ± 0.92	3.93 ± 1.08	0.04

Data are presented as mean ± SD, except BMI, hip, glucose, insulin, HOMA-IR and HbA1c that are presented as median (lower limit–upper limit)

*BMI* body mass index, *WHR* waist to hip ratio, *BAI* body adiposity index, *HOMA ß* homeostasis model assessment ß, *HOMA IR* homeostasis model assessment insulin resistance, *QUICKI* quantitative insulin sensitivity check inbox, *DDP4* Dipeptidyl peptidase-4

Additional analyses were performed to investigate whether the diagnosis of diabetes would impact in DPP4 levels. Eight patients with FPLD2 without diabetes were compared to the healthy control group and they presented significantly higher levels of DDP4 (5.1 ± 0.7 vs 3.9 ± 1.0; p = 0.022). Within FPLD2 group, patients with diabetes (n = 6) were compared to patients without diabetes (n = 8). No difference in DPP4 was found in these patients (4.5 ± 1.1 vs 5.1 ± 0.7; p = 0.28).

**Fig. 1:**
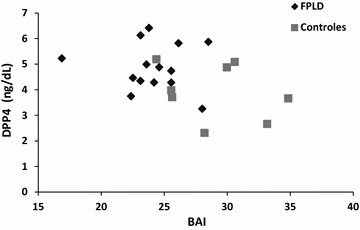
Correlation between dipeptidyl peptidase-4 (DPP4) levels and body fat distribution, measured by body adiposity index (BAI; r = −0.38; p = 0.072)

Correlation analysis was used to determine how anthropometric measurements and metabolic variables correlated with DPP4 levels. In our sample, no correlation could be demonstrated between DPP4 and hip (r = −0.29; p = 0.18), WHR (r = 0.03; p = 0.89), insulin (r = 0.16; p = 0.49), HOMA-β (r = 0.27; p = 0.23), HOMA-IR (r = 0.13; p = 0.56), QUICKI (r = −0.19; p = 0.40), HDL Cholesterol (r = −0.14; p = 0.51) and triglycerides (r = 0.30; p = 0.17). A trend toward an inverse statistical significance was observed between DPP4 and BAI (r = −0.38; p = 0.072; Fig. 1).

Body fat was also evaluated using DXA in nine patients with FPLD2. In these patients, a significant correlation was found between DPP4 levels and percentage of total body fat and (r = 0.86; p = 0.0025) and android fat (r = 0.78; p = 0.014).

## Discussion

Lipodistrophic syndromes are characterized by scarcity of subcutaneous adipose tissue in a generalized or localized fashion [7]. We have previously characterized body fat distribution by DXA in patients with generalized and partial lipodystrophy and have demonstrated that a central to peripheral fat ratio, named fat mass ratio (FMR) [10, 11] and BAI [12], may be useful to investigate fat distribution in these patients [10–12]. Our main results were that individuals with FPLD2 do have increased levels of DDP4 and that these levels might correlate with body fat distribution measured by DXA and estimated by BAI.

As stated above, it has been previously demonstrated that BAI inversely correlates with FMR in patients with FLPD2 [12] suggesting, at least in this population, that lower BAI scores may also reflect higher central-to-peripheral fat ratio (as a main anthropometric measure in BAI equation is hip circumference). In the present study, patients with FPLD2 had lower BAI and increased levels of DDP4. It has also been demonstrated a trend toward significance in the inverse correlation between BAI and DPP4 and that body fat content correlates with DPP4 levels, at least in patients with FPLD2. Taken together, these findings support the concept of DDP4 as an adipokine related to central fat. Indeed, more recently it was demonstrated that DPP4 is expressed in subcutaneous adipose tissue (SAT) as well as in visceral adipose tissue (VAT), with VAT exhibiting higher expression [5]. Ex vivo release of DPP4 from adipose explants was higher in VAT than SAT. Furthermore, circulating DPP4 correlated with the amount of VAT and, for the same BMI, insulin-resistant obese patients had higher DPP4 levels than insulin-sensitive obese patients [5]. Tanaka et al. [13] had previously demonstrated that serum DPP4 levels were positively correlated with visceral fat area (evaluated with computed tomography scan) in 135 men with type 2 diabetes. In their study, the positive association of the adipokine with visceral fat area remains unaffected even after adjusting for various parameters as average duration of diabetes, HbA1c and BMI, reinforcing an important correlation of body fat distribution with DPP4 levels.

Other adipokines have already been studied in FPLD2. Wong et al. [14] demonstrated that adiponectin, leptin and interleukin 6 (IL-6) are decreased, unlike the tumor necrosis factor-α (TNF-α) that is increased. These authors suggested that these changes are important determinants of IR. We have previously showed that RBP4 and leptin were decreased in FPLD2 and the later negatively correlated with WHR, what supported its relationship with the amount and distribution of body fat, but not with IR [6]. In the same way, the present data suggest that DPP4 can be a marker of adiposity and fat distribution, but does not associate with IR.

It is possible that liver fat deposition is a more plausible explanation for the correlation between these adipokines and IR. A study in patients with nonalcoholic steatohepatitis (NASH) showed an increase in serum activity of DPP4 [15]. Furthermore, the intensity of immunofixation of DPP4 in liver and its serum activity correlated with the histological grade of steatosis and NASH. Firneisz et al. [16] confirmed the increased activity of DPP4 in non-diabetic and diabetic patients with fatty liver disease. Unfortunately, the severity of liver disease was not accessed in this study and further studies are necessary to investigate this hypothesis.

An increased DDP4 concentration in patients with FPLD2 may have important implications for the physiopathology and treatment of diabetes in this population. Although no study has already been performed to investigate the impact of DDP4 inhibitors and/or GLP-1 agonists/analogs in lipodystrophy, it seems reasonable to suppose that they might be useful in these individuals, in a way that resembles its efficacy in type 2 diabetes and obesity.

Our study has some limitations. First, only a small number of individuals were included in each group. The small size our sample might help justifying some of our findings, including the lack of association between DPP4 levels and markers of insulin resistance, for instance. Second, DXA was only performed in a limited number of patients in the FLPD2 group. However, the strong relationship found between DPP4 levels and android fat, taken together with BAI, strengths the findings of our study.

## Conclusions

In conclusion, patients with FPLD2 exhibit an increase in DDP4 levels in comparison to a healthy control group. The increase in the levels of this enzyme does not seem to be related to the diagnosis of diabetes and might be associated with an increase in central fat (estimated using BAI and measured using DXA). These results might be used to reinforce the concept that DDP4 is an adipokine related to central fat distribution. Further studies are necessary to clarify the role of DPP4 inhibitors in the treatment of lipodystrophic patients.

## Abbreviations

DPP4: dipeptidyl peptidase-4
FPLD2: familial partial lipodystrophy type 2
BAI: body adiposity index
FMR: fat mass ratio
GLP-1: glucagon-like peptide-1
GIP: gastric inhibitory polypeptide
DXA: dual energy X-ray assessment
RBP-4: retinol binding protein 4
